# *Lacticaseibacillus paracasei* HP7 Improves Gastric Emptying by Modulating Digestive Factors in a Loperamide-Induced Functional Dyspepsia Mouse Model

**DOI:** 10.4014/jmb.2412.12035

**Published:** 2025-03-11

**Authors:** Ji-Woong Jeong, Daehyeop Lee, Hyeonji Kim, Hyeonjun Gwon, Kippeum Lee, Joo-Yun Kim, Jae-Jung Shim, Jae-Hwan Lee

**Affiliations:** R&BD Center, hy Co., Ltd., Yongin-si 17086, Republic of Korea

**Keywords:** *Lacticaseibacillus paracasei* HP7, functional dyspepsia, gastric emptying, gastrointestinal motility, digestive factor

## Abstract

Functional dyspepsia (FD) is a condition characterized by persistent indigestion symptoms without a clear underlying cause. We investigated the effects on FD of *Lacticaseibacillus paracasei* HP7 (HP7), which was isolated from kimchi and is known to inhibit *Helicobacter pylori*. In a mouse model of loperamide-induced FD, HP7 administration significantly improved gastrointestinal (GI) motility and gastric emptying, as demonstrated by increased charcoal movement in the GI tract, decreased stomach weight, and the amount of remaining phenol red solution. HP7 administration significantly enhanced peristalsis by upregulating the expression of smooth muscle contraction-related genes, such as the 5HT4 receptor, anoctamin-1, ryanodine receptor 3, and smooth muscle myosin light-chain kinase. In addition, digestive factors, including GI regulatory hormones such as gastrin, gastric inhibitory peptide, and peptide YY, and the activity of digestive enzymes, such as amylase, trypsin, and lipase, were restored to normal levels. These results indicate that HP7 is a promising probiotic strain to alleviate FD symptoms by modulating peristalsis and digestive factors.

## Introduction

Functional dyspepsia (FD) is one of the most common functional gastrointestinal (GI) diseases, affecting 10–30% of adults and 3.5–27% of children worldwide [1]. FD is divided into two diagnostic categories of meal-related dyspeptic symptoms based on the Rome IV criteria: postprandial distress syndrome (PDS), characterized by postprandial fullness and early satiation, and epigastric pain syndrome (EPS), characterized by epigastric pain and burning. In patients with FD, these symptoms persist for at least 1–3 days per week for > 3 months [2]. Recurrent and prolonged symptoms contribute to poor quality of life and economic burden [3]. Despite its high prevalence and disease burden, the pathophysiology of FD remains poorly understood. Previous studies have explored several mechanisms through impaired gastric accommodation [4], delayed gastric emptying [5], visceral hypersensitivity [6], and *Helicobacter pylori* infection [7]. FD can be treated with proton pump inhibitors, serotonin (5-hydroxytryptamine, 5-HT) receptor agonists, the eradicating drugs against *H. pylori*, prokinetic drugs, and psychotropic agents [8]. Nonetheless, the efficacy of these pharmacological therapies remains unsatisfactory, and many patients with FD are refractory to conventional pharmacological treatments [9]. This necessitates healthy treatments to alleviate FD.

Probiotics are beneficial for digestive health and effective in treating irritable bowel syndrome (IBS). The ameliorative effect of probiotics on IBS is potentially attributed to amplifying intestinal tight junctions and stabilizing permeability [10, 11]. In addition to their role in IBS, probiotics have demonstrated potential in improving symptoms associated with *H. pylori*-related dyspepsia and FD [12, 13]. The proposed mechanism for these effects includes the ability of probiotics to inhibit *H. pylori*, a common pathogen associated with gastric discomfort [14]. *H. pylori* is one of the contributing factors to FD symptoms; however, other factors, such as gastric motility and digestive enzyme function, also play significant roles in FD [15, 16]. Despite the growing evidence supporting the use of probiotics in treating GI disorders, the mechanisms by which they influence gastric motility and overall digestion remain unclear [17].

*Lacticaseibacillus paracasei* HP7, isolated from kimchi, is a probiotic strain capable of preventing *H. pylori* infection by reducing the invasion of *H. pylori* into gastric epithelial cells [18]. In this study, we investigated the effect of HP7 on gastric motility in the absence of *H. pylori* using a mouse model of loperamide-induced FD and assessed the potential of HP7 to improve FD symptoms (particularly PDS) by measuring its effects on digestive enzyme activity and peristalsis-related hormone secretion.

## Materials and Methods

### Bacterial Strain

*L. paracasei* HP7 (HP7) was stored as frozen stock in de Man-Rogosa-Sharpe broth (Difco Laboratories, USA) containing 20% (v/v) glycerol at -80°C and cultured at 35°C for 24 h in MRS broth. Cultured HP7 cells were centrifuged at 3,000 ×*g* for 15 min. The supernatants were discarded, and the cell pellets were washed twice with sterile phosphate-buffered saline (PBS). The cell pellets were collected and resuspended in PBS.

### Animal Study Design

Six-week-old male BALB/c mice, purchased from Dooyeol Biotech (Republic of Korea ), were maintained in a controlled environment (temperature 20–22°C, humidity 40–60%, 12 h light/dark cycle) and fed a rodent diet (crude protein 18.4%, fat 6.5%, carbohydrates 44.2%, crude fiber 3.8%, neutral detergent fiber 14.7%, and ash 5.5%; Envigo, USA) for 1 week for acclimation.

Next, the mice were randomly divided into five groups: non-treatment (normal), loperamide (10 mg/kg, control), loperamide + mosapride (3 mg/kg/day, mosapride, as a positive control), loperamide + *L. paracasei* HP7 1×10^8^ CFU/kg/day, and loperamide + *L. paracasei* HP7 1 × 10^9^ CFU/kg/day. To evaluate GI transit rate, six mice were used per group, while in the gastric emptying experiment, nine mice were used per group.

The normal and control groups were orally administered saline, while the mosapride and HP7 groups were orally administered each sample daily for 4 weeks. On the final day of the experiment, the mice were fasted for 20 h, and all, except those in the normal group, were administered a single intraperitoneal injection of loperamide. After 30 min, the mice were euthanized using CO_2_ gas in a chamber. Blood, stomach, duodenum, and small intestine samples were extracted for analysis. Blood samples were centrifuged at 3,000 ×*g* for 20 min to separate the serum. Tissues and separated serum samples were stored at -80°C.

The study protocol was conducted according to the guidelines of hy Co., Ltd. (Republic of Korea ) and approved by the Institutional Animal Care and Use Committee of hy Co., Ltd. (Approval no. AEC-2024-0002-Y, AEC-2024-0007-Y).

### Determination of GI Transit Rate Using Charcoal Diet

To evaluate the GI transit rate, the mice were orally administered 5% charcoal dissolved in 10% Arabic gum (200 μl/mouse) after 30 min of loperamide injection. After 30 min, the mice were sacrificed, and GI transit was determined by measuring the distance of charcoal transit from the pylorus to the cecum [19, 20].

### Determination of Gastric Emptying and Stomach Weight Using Phenol Red

The mice were fasted for 20 h with access to tap water. After 30 min of loperamide treatment, all the mice were orally administered phenol red solution (500 μl/mouse) prepared by adding 0.05% phenol red dissolved in distilled water containing 1.5% sodium carboxymethyl cellulose. Thirty minutes following phenol red solution administration, the mice were sacrificed, and their stomachs were immediately removed and weighed.

To determine gastric emptying, the stomachs were homogenized in 5 ml of 0.1 N sodium hydroxide solution and 20% (w/v) trichloroacetic acid (0.5 ml). The solutions were centrifuged at 700 ×*g* for 20 min, and 1 ml of the resulting supernatants was added to 4 ml of 0.5 N sodium hydroxide. The absorbance of the solution was measured at 560 nm using a BioTek Synergy HTX multimode reader (Agilent).

The gastric emptying rates were calculated according to the following formula:

$$\text{Gastric emptying (\%)}=\frac{1-X}{Y}\times 100,$$

where X is the absorbance of phenol red solution remaining in the stomach, and Y is the absorbance of naïve phenol red mixed with sodium hydroxide.

### Extract of Total RNA and Measurement of Smooth Muscle Contraction-Related Gene Expression by Quantitative Real-Time Polymerase Chain Reaction

Total RNA was extracted from the stomach using an Easy-Spin Total RNA Extraction Kit (iNtRON Biotechnology, Republic of Korea), and cDNA was synthesized using an Omniscript Reverse Transcription Kit (Qiagen, Germany). Synthesized cDNA was analyzed using quantitative real-time polymerase chain reaction (RT-qPCR) with TaqMan Gene Expression Assays (Applied Biosystems). The genes used in this study were as follows: Glyceraldehyde-3-phosphate dehydrogenase (GAPDH, Mm99999915_g1), 5-hydroxytryptamine (serotonin) receptor 4 (5HT4R, Mm00434129_m1), anoctamin-1 (ANO1, Mm00724407_m1), ryanodine receptor 3 (Ryr3, Mm01328421_m1), and smooth muscle cell myosin light-chain kinase (smMLCK, Mm00653039_m1). Relative mRNA expression data were normalized to GAPDH.

### Determination of GI Regulatory Hormones and Digestive Enzyme Activity

The prepared serum was used to measure the digestive hormone secretion. Commercial ELISA kits (CUSABIO, USA) were used to evaluate the levels of gastrin (GAS, CSB-E12924), gastric inhibitory peptide (GIP, CSB-E08486m), and peptide YY (PYY, CSB-EL019128MO) secretion, according to the manufacturer’s instructions. The absorbance was measured at 450 nm using a BioTek^®^ Synergy HTX multimode reader.

The small intestine tissue (100 mg) was homogenized in 1 ml of PBS. The homogenate was centrifuged at 3,000 ×*g* for 20 min, and the supernatant was collected to evaluate amylase, trypsin, and lipase activities. Digestive enzyme activity was detected using the Amylase Assay Kit (ab102523, Abcam, UK), Trypsin Activity Assay Kit (ab102531, Abcam), and Lipase Assay Kit (ab102524, Abcam), according to the manufacturer’s instructions.

### Statistical Analysis

The data are expressed as mean ± SD. Statistical significance was analyzed using an unpaired Student’s *t*-test with SPSS version 26.0 (IBM Corp., USA).

## Results

### Effects of HP7 on GI Charcoal Transit Ratio

**Fig. 1:**
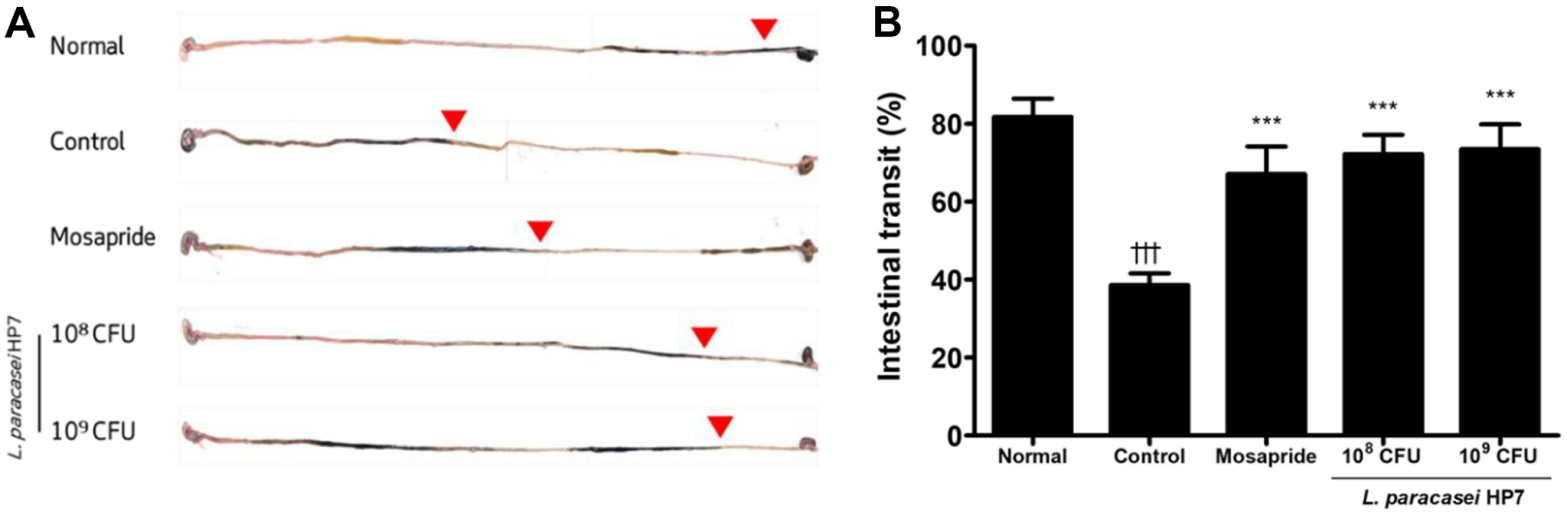
Effects of *Lacticaseibacillus paracasei* HP7 on gastrointestinal motility. After charcoal diet, (A) the moved distance from pylorus to cecum is indicated with red arrows and (B) quantified. The data are presented as the mean ± SD. Significant differences were expressed as †††*p* < 0.001 vs. normal, **p < 0.005 vs. control, ***p < 0.001 vs. control.

The intestinal transit was expressed as the ratio of the passage distance by charcoal to the total length from the pylorus to the cecum. Loperamide injection markedly diminished intestinal transit (Fig. 1A) compared with the normal group. The intestinal transit rate was significantly reduced (*p* < 0.001) to approximately 38% in the loperamide group compared with 81% in the normal group (Fig. 1B).

Administration of mosapride or HP7 significantly recovered the decreased transit rate. Treatment with HP7 showed similar effects in improving intestinal transit even when different doses were administered.

### HP7 Reverses Gastric Emptying Delayed by Loperamide

**Fig. 2:**
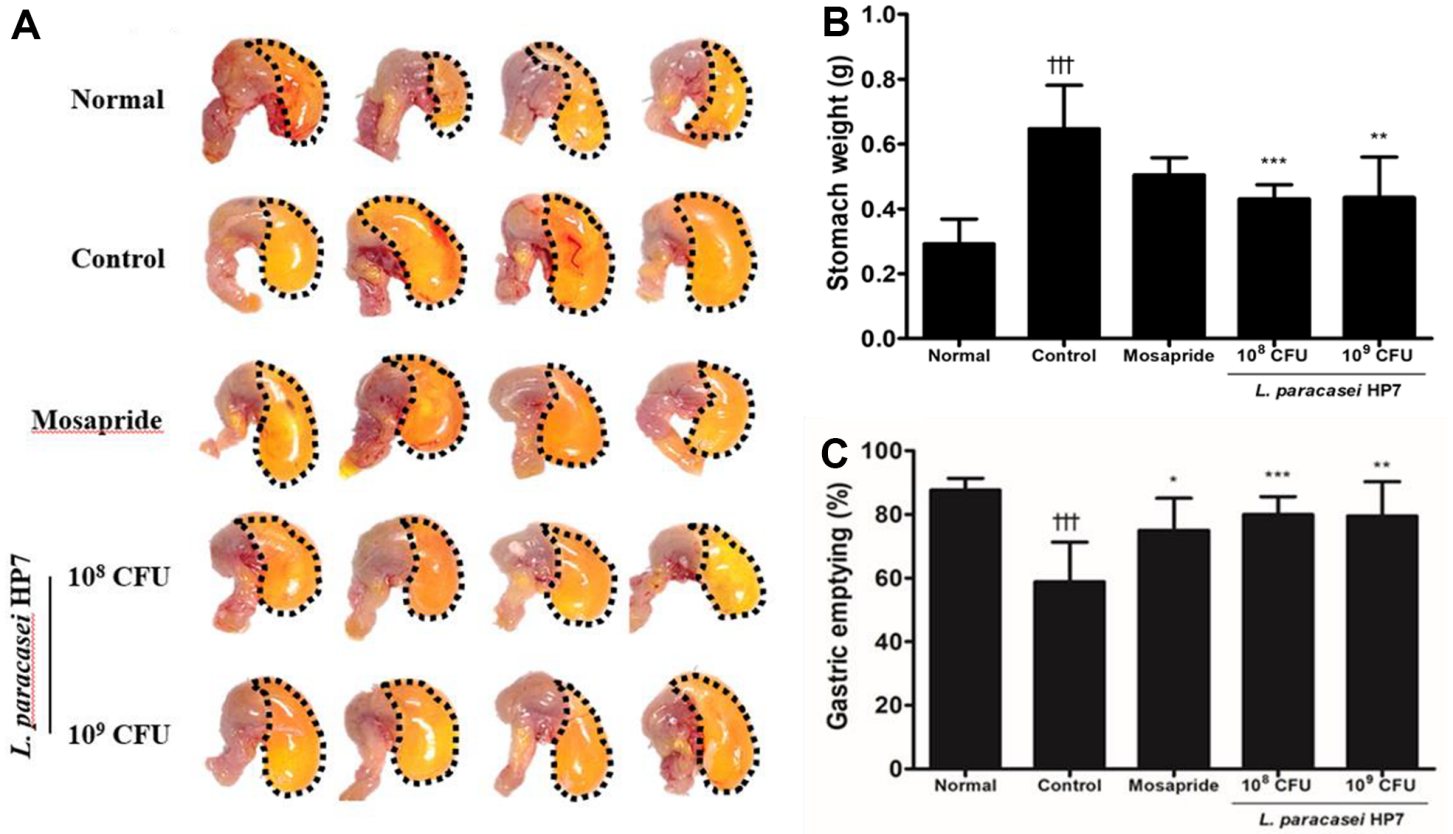
Effects of *Lacticaseibacillus paracasei* HP7 on gastric emptying. After phenol red solution administration, (**A**) visually observed stomachs, (**B**) stomach weight, and (**C**) gastric emptying was evaluated. Data are presented as the mean ± SD. Significant differences were expressed as †††*p* < 0.001 vs. normal, *p < 0.05 vs. control, **p < 0.005 vs. control, ***p < 0.001 vs. control.

**Fig. 3:**
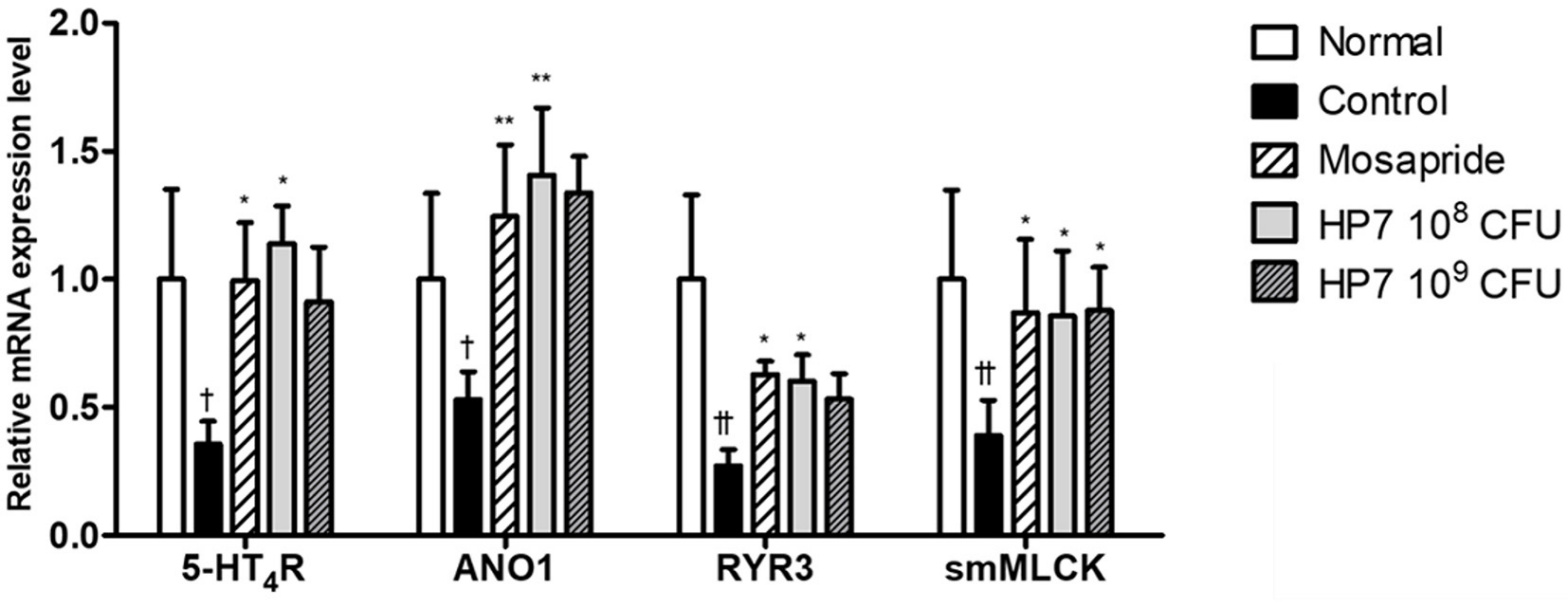
Expression of smooth muscle-related gene in the stomach affected by *Lacticaseibacillus paracasei* HP7 in loperamide-induced mouse model. Data are presented as the mean ± SD. Significant differences were expressed as †*p* < 0.05 vs. normal, ††*p* < 0.005 vs. normal, *p < 0.05 vs. control, **p < 0.005 vs. control.

In our study, loperamide delayed gastric emptying, compared with the normal group. The stomach was filled with phenol red (Fig. 2A), significantly increasing the stomach weight (*p* < 0.001; Fig. 2B). This finding is supported by the significantly (*p* < 0.001) decreased gastric emptying ratio, evaluated by quantifying the remaining phenol red in the stomach (Fig. 3B). The HP7-treated group, on the contrary, showed a visually smaller stomach, resulting in a significant decrease in stomach weight. Furthermore, the gastric emptying ratio was significantly recovered following HP7 administration. Treatment with HP7 demonstrated comparable effects in enhancing gastric emptying at different doses. The mosapride-treated group showed a tendency similar to that of the HP7-treated group.

### Effects of HP7 on mRNA Levels of Smooth Muscle Contraction-Related Genes in Gastric Antrum

The expression of smooth muscle contraction-related genes, including 5-HT_4_R, ANO1, RYR3, and smMLCK, was reduced following loperamide injection. This indicates that these gene expression changes may be responsible for the reduction in gastric antrum. Mosapride treatment exerted a positive effect on the expression of these four genes, significantly upregulating mRNA expression. Additionally, the groups treated with HP7 restored the mRNA expression of genes related to smooth muscle contraction to levels comparable to or greater than those observed in the mosapride-treated group (Fig. 3).

### Effects of HP7 on GI Regulatory Hormones and Digestive Enzyme Activities

**Fig. 4:**
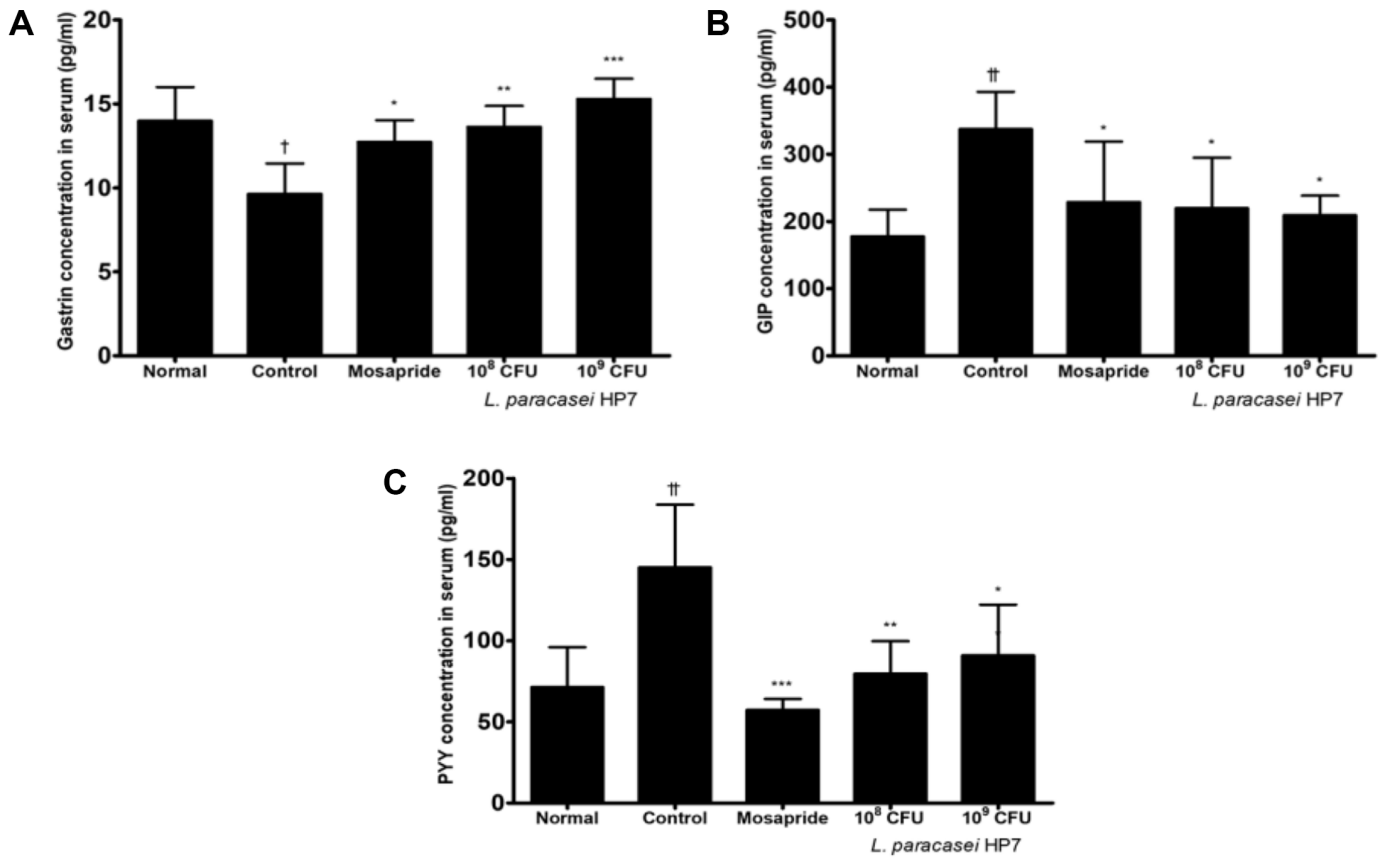
Changes in concentration of gastrointestinal regulatory hormones (A) gastrin, (B) GIP, and (C) PYY in serum following treatment with *Lacticaseibacillus paracasei* HP7 in a loperamide-induced mouse model. Data are presented as the mean ± SD. Significant differences were expressed as †*p* < 0.05 vs. normal, ††*p* < 0.005 vs. normal, *p < 0.05 vs. control, ***p < 0.001 vs. control.

HP7 significantly altered the serum levels of GI regulatory hormones, such as GAS, GIP, and PYY (Fig. 4). The serum concentration of GAS, which is associated with improved GI motility, reduced from 13.9 to 9.6 pg/ml following loperamide injection, but was restored to normal levels at 13.9 and 13.6 pg/ml when administered with mosapride or HP7 (Fig. 4A), respectively. The concentration of GIP, a digestive hormone, was increased with loperamide injection up to 332 pg/ml compared with the normal group, and the concentration was decreased following treatment with mosapride or HP7 (Fig. 4B). Moreover, the serum concentration of PYY also increased from 71.3 pg/ml in the normal group to 145.1 pg/ml in the control group but decreased following treatment with mosapride or HP7 (Fig. 4C).

**Fig. 5:**
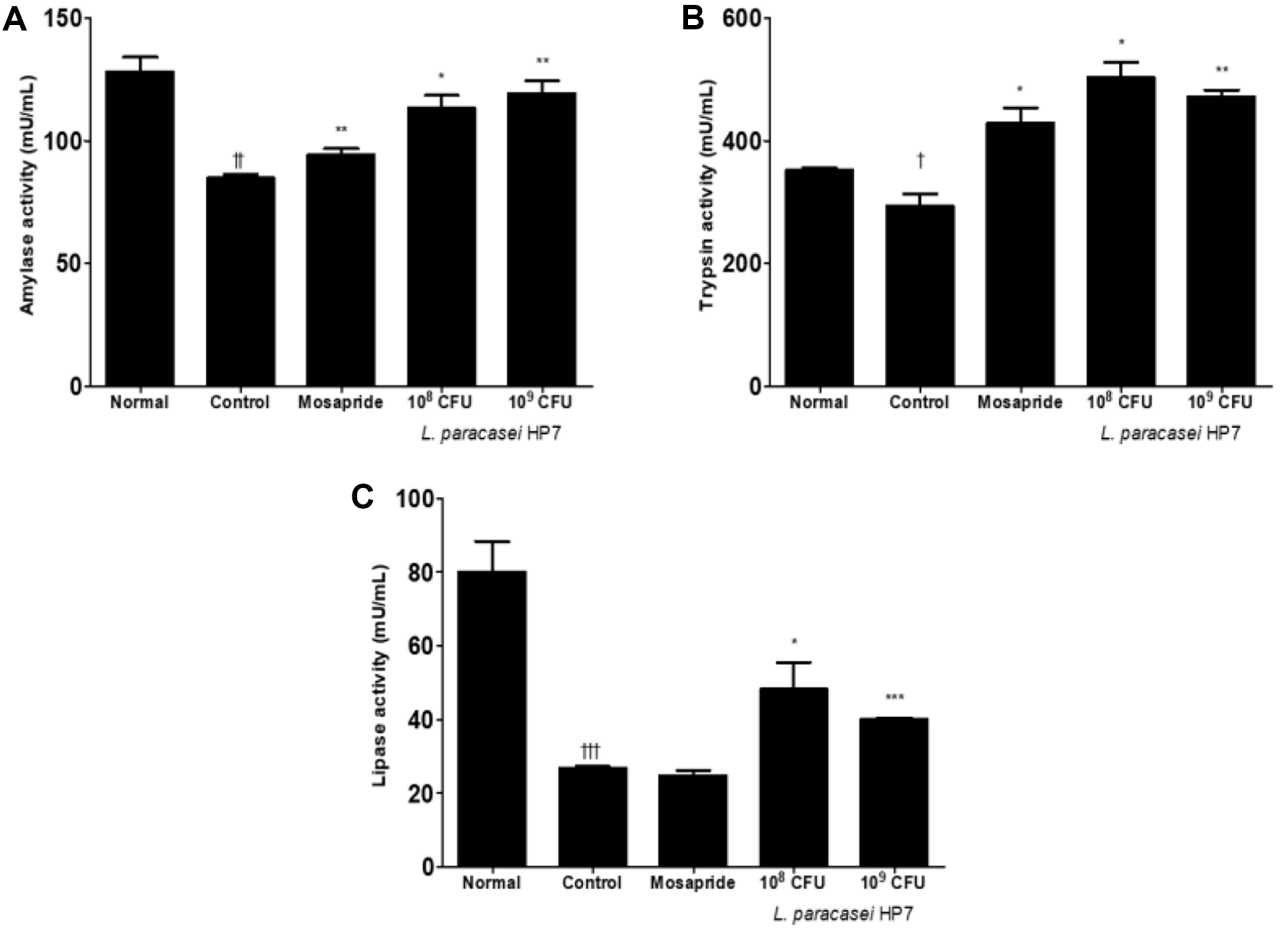
Changes on digestive enzyme activity (A) amylase, (B) trypsin, and (C) lipase in small intestine following treatment with *Lacticaseibacillus paracasei* HP7 in a loperamide-induced mouse model. Data are presented as the mean ± SD. Significant differences were expressed as †*p* < 0.05 vs. normal, ††*p* < 0.005 vs. normal, †††*p* < 0.001 vs. normal, *p < 0.05 vs. control, *p < 0.005 vs. control, ***p < 0.001 vs. control.

The activities of digestive enzymes such as α-amylase, trypsin, and lipase are closely associated with digestive function [20]. Loperamide significantly downregulates the activity of these enzymes. Small intestine α-amylase activity, reduced from 128 to 84 mU/ml following loperamide injection, was significantly recovered to 113 and 119 mU/ml following HP7 administration (Fig. 5A). Trypsin activity was also reduced by loperamide and significantly increased following treatment with mosapride or HP7 (Fig. 5B). The lipase activity was 80 mU/ml in the normal group and decreased to 26 mU/ml (a 33% reduction) in the loperamide-injected group. Specifically, mosapride did not affect lipase activity, although it was significantly improved in the HP7 treatment group (*p* < 0.05; Fig. 5C). These results show that HP7 improves GI regulatory hormones and digestive enzyme activity in a mouse model of loperamide-induced FD.

## Discussion

The pathophysiology of FD is complex and may involve multiple etiologies. Nonetheless, abnormalities in gastric motility are a primary cause. Research on the brain-gut axis has indicated that various stress-induced symptoms may result from motor dysfunction and visceral hypersensitivity mediated by brain-gut interactions. Additionally, *H. pylori*-related FD and delayed gastric emptying have been reported, emphasizing the relationship between gastric motility and abnormalities in GI mucosal immunity [21]. In our earlier research, the probiotic strain *L. paracasei* HP7 was found to inhibit *H. pylori* infection by decreasing its invasion into gastric epithelial cells, demonstrated through both in vitro and in vivo studies [18]. HP7 meets the antibiotic resistance standards set by the European Food Safety Authority (Table S1) and has been confirmed to have excellent digestive stability and intestinal cell adhesion (Figs. S1, S2). In addition, this strain did not show hemolytic reactions on blood agar plate (Fig. S3), and its genetic information was analyzed through whole genome analysis, confirming its potential as a probiotic (Fig. S4). Therefore, in this study, we verified the effect of HP7 on improving gastric health, especially FD symptoms, from the pharmacological and physiological perspective in an environment unrelated to *H. pylori* infection.

A loperamide-induced mouse model was used to evaluate the in vivo efficacy of HP7 in FD. Loperamide, a μ2-opioid receptor agonist, suppresses the activity of the GI myenteric plexus, which decreases the tone of the circular and longitudinal smooth muscles of the GI tract [20]. In recent studies, loperamide has been used to delay gastric emptying and slow GI motility in mouse models [19, 20, 22]. In this study, loperamide injection (10 mg/kg) delayed gastric emptying, as evidenced by the observed weight increase and the remaining phenol red solution in the stomach, consistent with previous findings. Delayed gastric emptying is a typical feature of FD [23], and our results suggest that loperamide injections induce FD effectively. Loperamide-induced delayed gastric emptying is significantly ameliorated by HP7 administration in a mouse model. Improved GI motility was demonstrated through increased charcoal movement distance in the GI tract, while improved gastric emptying was demonstrated by decreased stomach weight and the amount of remaining phenol red solution.

Administration of HP7 regulated the expression of four genes (*5-HT_4_R*, *ANO1*, *RYR3*, and *smMLCK*) in the stomach tissue, confirming the effect of HP7 discussed above. Moreover, 5-HT and its counterpart 5-HT_4_R are involved in regulating smooth muscle contraction activity [24], while 5-HT_4_R agonists, such as mosapride, are the primary treatment options for functional GI motility disorders [25].

In our study, HP7 or mosapride administration upregulated the expression of 5-HT_4_R in the stomach tissue of treated mice. The effect of HP7 on enhancing 5-HT receptor expression may be related to our previous finding indicating that HP7 increases the expression of brain-derived neurotrophic factor (BDNF) [26], a vital neutrophil factor in the GI tract that controls visceral sensation, motility, and intestinal barrier function and is observed at low levels in patients with FD [27]. Although the action of BDNF in the gut has not been fully characterized, endogenous BDNF appears to enhance peristalsis by increasing 5-HT levels [28]. Therefore, our results, along with those of previous studies [26], indicate that the interaction between serotonin, 5-HT, and BDNF may help to alleviate FD symptoms; however, further studies are warranted.

GI motility is related to the slow-wave potential induced by the interstitial cells of Cajals (ICCs) [29]. ICCs mediate the input from the GI motor nervous system to smooth muscles and play a crucial role in GI motility by generating spontaneous electrical slow waves that stimulate peristalsis [30]. Slow waves are generated by the spark of intracellular Ca^2+^ concentration changes in ICCs and rely on the functions of ANO1 and RYR3 [31, 32]. The generated waves deliver electrical signals to smooth muscle cells via gap junctions, which cause smooth muscle contraction via active smMLCK [20, 33]. Although the activation of ICCs was not evaluated in this study, our results imply that HP7 intake improves gastric emptying and GI motility by upregulating smooth muscle-related genes.

The effects of HP7 on GI regulatory hormones and digestive enzyme activity were evaluated. Excitatory GI regulatory peptides, such as GAS, and inhibitory GI regulatory peptides, such as PYY, are crucial to regulating GI functional disorders [34]. GAS is the peptide hormone secreted by G cells that strongly stimulates the secretion of gastric acid and pepsin; PYY is a typical appetite-suppressing hormone secreted by L cells, which inhibits gastric acid secretion and GI motility, inducing the feeling of satiety and reducing food intake [35, 36].

GIP, an enzyme involved in intestinal physiological functions, can influence the mucosal immune system, permeability, and enteric nervous system (ENS), and may contribute to the development of dyspepsia [37]. The decreased concentrations of GAS and increased concentrations of PYY and GIP following loperamide injection were restored to normal levels following HP7 administration. Additionally, loperamide injection influenced the digestive enzymes, including α-amylase, trypsin, and lipase, which are directly related to GI digestive function [34]. In this study, loperamide injection decreased digestive enzyme activity, consistent with previous findings [34]. Enzyme activity was significantly increased by HP7 administration, suggesting that HP7 can improve FD by regulating the expression of GI regulatory hormones or digestive enzyme activity.

In conclusion, this study demonstrated the effect of *L. paracasei* HP7 in a mouse model. However, the results of this study have limitations in proving the underlying mechanism of HP7 administration’s effect on improving gastric emptying because only smooth muscle contraction genes or digestive factors were analyzed. Although the improved gastric emptying may be related to changes in the gut microbiota after probiotic intake, a limitation of this study is the lack of identifying any changes in the gut microbiota and its metabolites. Nonetheless, this study provides evidence supporting the health benefits of HP7 in improving FD symptoms, particularly PDS. As such, HP7 can potentially be used as a beneficial probiotic strain for the treatment and alleviation of FD.

## Supplemental Materials

Supplementary data for this paper are available on-line only at http://jmb.or.kr.
