# Supplementary material for: Lacticaseibacillus paracasei HP7 Improves Gastric Emptying by Modulating Digestive Factors in a Loperamide-Induced Functional Dyspepsia Mouse Model
Source: J Microbiol Biotechnol. 2025 Mar 11;35:e2412035. doi: 10.4014/jmb.2412.12035 (PMC11925751; doi:10.4014/jmb.2412.12035)
Supplement: Supplementary file 1 [file jmb-35-e2412035-supple.pdf]

J. Microbiol. Biotechnol. 2025. 35: e2412035

https://doi.org/10.4014/jmb.2412.12035

Supplementary Table and Figures

Probiotic properties of HP7

The minimum inhibitory concentrations (MICs, μg/mL) of eight antibiotics were determined according to the ISO 10932:2010 standard [1]. Epidemiological limit values were derived from the recommendations of the European Food Safety Authority (EFSA) [2] and all antibiotics were purchased from Sigma Aldrich. Hemolytic activity was measured by culturing for 48 hours on Columbia agar (KisanBio, SEOUL, Korea) containing 5% sheep blood. Hemolytic activity by β-hemolysin was determined by the presence or absence of a clear zone around the colony [1]. Gastrointestinal stability and intestinal colonization were determined with reference to previous studies. Gastrointestinal stability was confirmed by measuring the number of viable strains of the strain in a simulated gastrointestinal environment passing through the mouth, stomach, and small intestine [3], and intestinal colonization was confirmed by measuring the number of LAB attached to Caco-2, a human adenocarcinoma colon cell [3,4].

Whole genome analysis of HP7

The complete genome of HP7 was analyzed on the Illumina MiSeq (Illumina, Inc., San Diego, CA, USA) and PacBio RS II (Pacific Biosciences Inc., Menlo Park, CA, USA) platforms at CJ Biosciences (Seoul, Korea). Library construction was performed using the TruSeq DNA Library LT Kit for Illumina (Illumina, San Diego, CA, USA) and the SMRTbell Template Preparation Kit for PacBio (100-938-900). Results were assembled by Unicycler v 0.4.3, and gene prediction and annotation were performed using CJ Bioscience's EzBioCloud database.

Table S1. Antibiotic resistance and minimum inhibitory concentrations (MIC).

Antibiotics	Cut-off Values* (mg/L)	MIC (mg/L)	Interpretation
Ampicillin	4	1	S
Vancomycin	n.r.	128	n.r.
Gentamycin	32	2	S
Kanamycin	64	64	S
Streptomycin	64	32	S
Erythromycin	1	0.5	S
Clindamycin	1	0.125	S
Tetracycline	4	1	S
Chloramphenicol	4	4	S

* Cut-off values published by the European Committee on Antimicrobial Susceptibility Testing (EUCAST, http://www.eucast.org/), EFSA; n.r, not required; s, sensitive

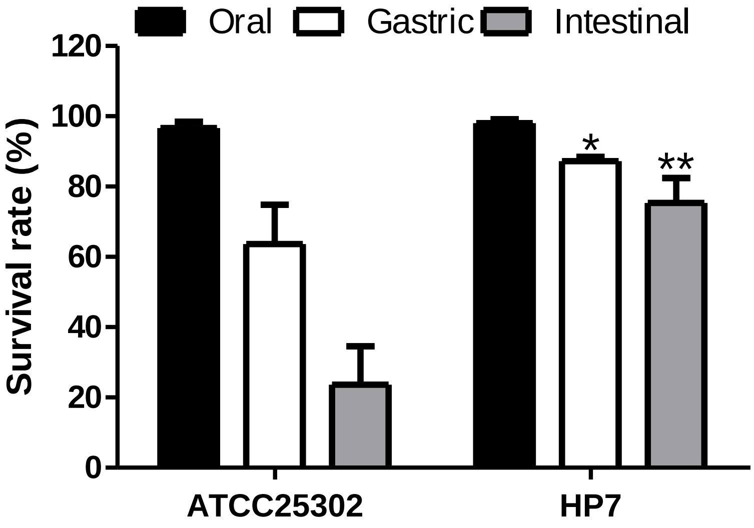

Fig. S1. Survival rate of lactic acid bacteria (LAB) strains under simulated gastrointestinal tract conditions. Data are represented as mean ± SD of three independent experiments. *$p < 0.05$ and **$p < 0.01$ compared with ATCC25302.

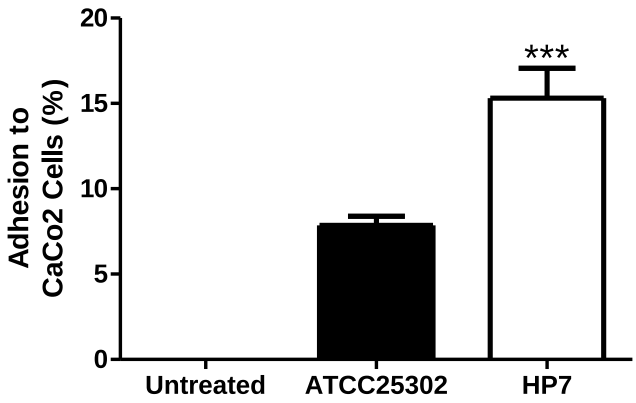

Fig. S2. Adhesion ability of LAB strains to intestinal epithelial cells in co-culture conditions LAB were added to Caco-2 cells at a ratio of 10:1. For adhesion analysis, the adhesion rate was calculated based on the inoculum added to Caco-2 cells. Data are represented as mean ± SD of three independent experiments. ***$p < 0.001$ compared with ATCC25302.

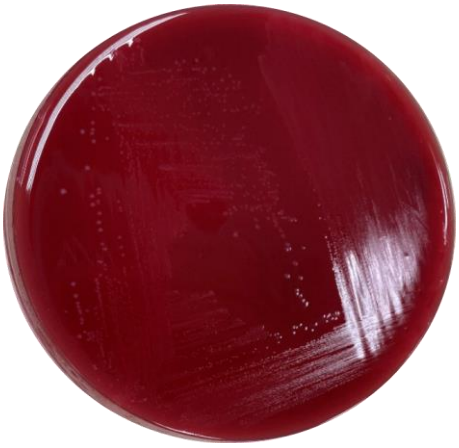

Fig. S3. Confirmation of β-hemolytic reaction of HP in blood agar plate. No β-hemolytic reaction.

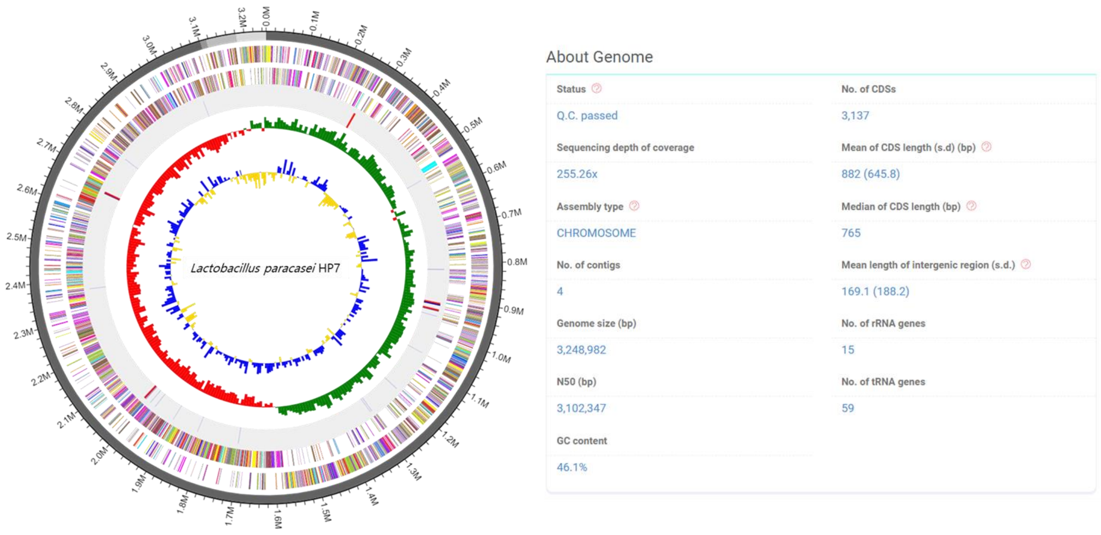

Fig. S4. Genomic map of the HP7 whole-genome. The total genome size of HP7 was 3,248,982bp, the CG ratio was 46.1%, and the coding sequence (CDS), which is the DNA region actually expressed as protein, was 3,137. The number of transfer RNA (tRNA), small molecule RNAs involved in protein synthesis through gene expression, was 59.

References

1. Aristimuño Ficoseco, C.; Mansilla, F.I.; Maldonado, N.C.; Miranda, H.; Fátima Nader-Macias, M.E.; Vignolo, G.M. Safety and growth optimization of lactic acid bacteria isolated from feedlot cattle for probiotic formula design. *Front. Microbiol.* **2018**, *9*, 2220.

2. Guidance on the assessment of bacterial susceptibility to antimicrobials of human and veterinary importance. *EFSA J.* 2012, 10, 2740.

3. Probiotic potential of a novel vitamin B2-overproducing Lactobacillus plantarum strain, HY7715, isolated from Kimchi. *Appl. Sci.* 2021, 11:5765. doi: 10.3390/app11135765.

4. Probiotic Properties and Safety Evaluation of Lactobacillus plantarum HY7718 with Superior Storage Stability Isolated from Fermented Squid. *Microorganisms*. 2023, 8;11(9):2254. doi: 10.3390/microorganisms11092254
